# Genomics and Prognosis Analysis of Epithelial-Mesenchymal Transition in Glioma

**DOI:** 10.3389/fonc.2020.00183

**Published:** 2020-02-21

**Authors:** Chuming Tao, Kai Huang, Jin Shi, Qing Hu, Kuangxun Li, Xingen Zhu

**Affiliations:** ^1^Department of Neurosurgery, The Second Affiliated Hospital of Nanchang University, Nanchang, China; ^2^Scientific Research Center, East China Institute of Digital Medical Engineering, Shangrao, China; ^3^Queen Mary School, Jiangxi Medical College, Nanchang University, Nanchang, China

**Keywords:** gene expression profile, glioma, epithelial-mesenchymal transition epigenetics, prognostic signature, overall survival

## Abstract

**Background:** Epithelial-mesenchymal transition (EMT) is regulated by induction factors, transcription factor families and an array of signaling pathways genes, and has been implicated in the invasion and progression of gliomas.

**Methods:** We obtained the Clinicopathological data sets from Chinese Glioma Genome Atlas (CGGA). The “limma” package was used to analyze the expression of EMT-related genes in gliomas with different pathological characteristics. We used the “ConsensusClusterPlus” package to divide gliomas into two groups to study their correlation with glioma malignancy. The least absolute shrinkage and selection operator (LASSO) Cox regression was applied to select seven prognosis-associated genes to build the risk signature, and the coefficients obtained from the LASSO algorithm were used to calculate the risk score which we applied to determine the prognostic value of the risk signature. Univariate and multivariate Cox regression analyses were used to determine whether the risk signature is an independent prognostic indicator.

**Results:** We analyzed the differentially expressed 22 common epithelial-mesenchymal transition-associated genes in 508 gliomas graded by different clinicopathological features. Two glioma subgroups (EM1/2) were identified by consistent clustering of the proteins, of which the EM1 subgroup had a better prognosis than the EM2 subgroup, and the EM2 group was associated with cancer migration and proliferation. Significant enrichment analysis revealed that EMT-related transcriptional regulators and signaling pathways genes were highly related to glioma malignancies. Seven EMT-related genes were used to derive risk scores, which served as independent prognostic markers and prediction factors for the clinicopathological features of glioma. And we found the overall survival (OS) was significantly different between the low- and high-risk groups, the ROC curve indicated that the risk score can predict survival rates for glioma patients.

**Conclusion:** EMT-related induction factors, transcriptional regulators and signaling pathways genes are important players in the malignant progression of glioma and may help in decision making regarding the choice of prognosis assessment and provide us clues to understand EMT epigenetic modification in glioma.

## Introduction

Glioblastoma (GBM) is the most malignant and most aggressive tumor of the brain (1). If left without intervention, the median survival of glioblastoma multiforme is 3 months (2). About 88% of GBM patients die within 1 year, the 5-year survival rate is <5% (3). Thus, GBM is considered to be a challenging malignancy globally.

Epithelial-mesenchymal transition (EMT) is characterized by the interaction of polarized epithelial cells with the basement membrane. The basal membrane of these cells exhibits a variety of cellular processes that makes them manifest different mesenchymal cell phenotypes, including invasiveness and migration, anti-apoptosis and high levels of ECM (4). It is also essential in wound healing, stem cell characteristics and in tissue fibrosis which determines cancer deterioration (5). This indicates that EMT contributes to the invasiveness of glioblastoma.

The transformation of EMT is triggered by various induction factors and transcription factors. The induction factors include epidermal growth factor (EGF), hepatocyte growth factor (HGF), fibroblast growth factor (FGF), Platelet-derived growth factor (PDGF), hypoxia-inducible factor (HIF), transforming growth factor (TGF) superfamily, and Insulin growth factor 1 (IGF1) (6, 7). When the growth factor interacts with the RTK (receptor tyrosine kinase), it phosphorylates the tyrosine residue of RTK itself, thereby activating the downstream PI3K/AKT pathway, MAPK pathway, SRC pathway, etc., which induces EMT in cells. This also leads to the activation of transcription factors, such as basic helix-loop-helix transcription factors, zinc-finger E-box-binding (ZEB) and SNAIL, which regulate transcription, translation, and post-translational levels of various molecules (5). Studies have shown that EMT transcriptional regulators, such as ZEB2, ZEB1, SNAI2, SNAI1, TWIST1, TWIST2, CDH1, and CDH2 are essential in promoting cell invasion, migration, proliferation, and angiogenesis (8–12). In the main signaling pathways, Wnt/β-Catenin, TGFβ, PI3K/Akt, ERK, CXCR4, BIRC5, CTNNB1, FZD4, HGF, EGFR, and other proteins enhance the expression of EMT markers and promote the migration and proliferation of glioblastoma (13–17).

EMT is determined to be closely related to glioma malignancies (18, 19). However, the expression profile of transcription factors and proteins associated with EMT and the diverse pathological features of gliomas have not been comprehensively investigated. In addition, their prognostic value in glioma is not known.

In this study, we used RNA sequencing data from Chinese Glioma Genome Atlas (CGGA) (*n* = 508) to systematically analyze the expression of 22 widely reported EMT-related induction factors, transcription factors and signaling pathways genes in glioma specimen. We provide data on the expression of each EMT-related genes in terms of different pathological characteristics. We found that transcription factors associated with EMT and the expression of signaling pathways genes are essential to the malignancy of glioma, and seven genes have prognostic value in glioma.

## Materials and Methods

### Data Collection

We obtained the Clinicopathological data sets from CGGA (*n* = 508) (http://www.cgga.org.cn/) and TCGA (*n* = 613) (http://cancergenome.nih.gov/). All the Clinicopathological information for the CGGA and TCGA datasets were shown in Figure S1.

### Select EMT Related Genes

To identify EMT related genes, we first compiled 22 EMT-related inducible factors, transcription factors and signaling pathways genes from published literature (18, 20). An array of protein lists and CGGA data sets were screened to obtain genes and RNA expression data. We then systematically compared the expressed EMT-associated transcription features and signaling pathways genes in gliomas with different clinicopathological features, including the IDH status in LGG and GBM, the1p19q status in LGG, and LGG with mIDH in CGGA dataset. The translational-level validation of EMT related genes was carried out using the ONCOMINE platform (www.oncomine.org) (21) and The Human Protein Atlas database (https://www.proteinatlas.org/) (22).

### Bioinformatics Analysis

To explore the function of gliomas' EMT related genes, we classified gliomas into various clusters. Use “ConsensusClusterPlus” (50 iterations, 80% resampling rate Pearson correlation, http://www.bioconductor.org/). R v3.5.1 R package PCA was applied to investigate gene expression arrays in the glioma groups.

### Annotation and Differential Analysis of EMT Related Genes

GO and KEGG pathway enrichment analyses were conducted using Annotation, Visualization, and Integrated Discovery databank (http://david.abcc.ncifcrf.gov/home.jsp) to functionally annotate genes that are differentially expressed in different groups. GSEA was performed to investigate the functions correlated with different subgroups of gliomas.

### Build Lasso Regression Model and Risk Survival Analysis

We screened the manifestation levels for the 22 genes in the CGGA data group using univariate Cox regression analysis. Thus, we identified 14 genes that were highly related to survival (*P* < 0.01). We chose these genes for more functional investigation and developed possible risk score using the LASSO Cox regression algorithm (23–26). Finally, minimum criteria defined the seven genes and their constants, choosing the perfect penalty parameter λ related to the minimum 10-fold cross validation inside the training set. Using the following formula, the risk score was calculated:

$$\begin{array}{l} Risk\;score=\overset{n}{\underset{i=1}{\sum }}\left(Coef_{i}*x_{i}\right) \end{array}$$

where Coef_i_ is coefficient, and xi is the z-score-transformed relative expression value of each selected gene. This was applied for each CGGA dataset's patient.

### Statistical Analysis

The expression of EMT related genes and their relationship with WHO grades were analyzed using one-way ANOVA. The *t*-test was applied to compare age, gender, IDH status, and 1p/19q codel status in glioma. Patient data were categorized into low and high-risk groups based on the median risk score as a cutoff value. Chi-square test was conducted to compare the distribution of clinical and molecular pathological features between the two risk groups. To compare glioma characteristics with different clinical pathology risk scores, a one-way ANOVA or *t*-test was conducted to compare risk scores for patients grouped by clinical or molecular pathology characteristics. Operating characteristic (ROC) curves were used to study the prediction efficiency of the risk signature, WHO grade and age for survival, EM1/2 groups, IDH-mutant status, and 1p/19q codeletion status. Univariate and multivariate Cox regression analyses were performed to determine prognostic values for risk scores as well as various clinical and molecular pathological features, including risk scores, age, gender, IDH status, and 1p/19q codel. The two-sided log-rank test Kaplan–Meier method was used to investigate the OS of the patients in the EM1/2 groups or in the high- and low-risk groups. All statistical analyses were conducted using R v3.5.1 (https://www.r-project.org/), SPSS 16.0 (SPSS Inc., Chicago, IL) and Prism 8 (GraphPad Software Inc., La Jolla, CA).

## Results

### Association of Expression Patterns of EMT Related Genes and Pathological Characteristics

**Figure 1:**
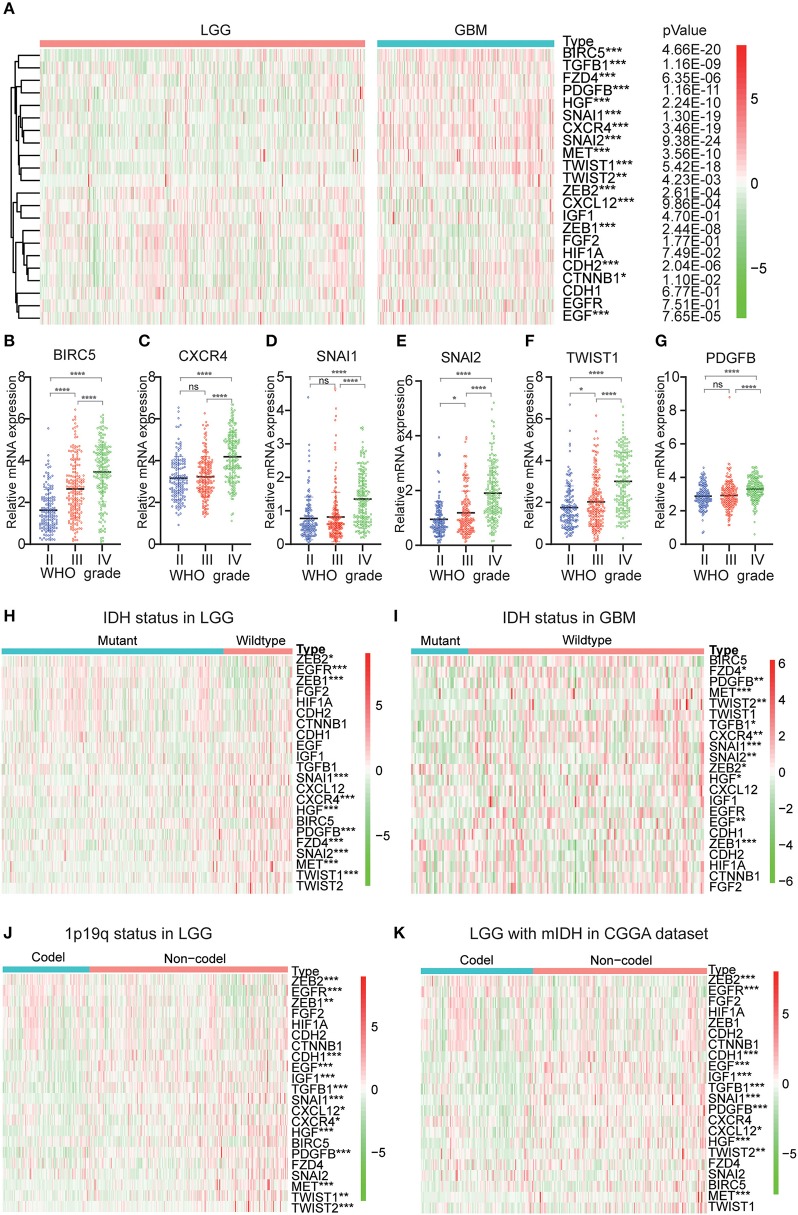
Expression of EMT related genes in gliomas with different clinicopathological features. **(A–G)** The expression levels of 22 EMT related genes in gliomas with different WHO grades. **(H,I)** The expression levels of EMT related genes in LGG and GBM with different IDH status. **(J)** The expression levels of EMT related genes in LGG with different 1p/19q codeletion status. *P<0.05, **P<0.01, ***P<0.001 and ****P<0.0001. **(K)** The expression levels of EMT related genes in IDH-mutant (mIDH) LGG with different 1p/19q codeletion status. *P<0.05, **P<0.01, ***P<0.001 and ****P< 0.0001.

Because EMT is determined to be closely related to glioma malignancies, we systematically studied the relationship between variables and the pathological characteristics of glioma, including WHO grade, IDH status, and 1p/19q codeletion status. Expression levels of transcription factors and signaling pathways genes and the WHO levels associated with each EMT variable are shown in the heatmap (Figures 1A–G). The heatmap reveals that the expression of most EMT induction factors, transcription factors, and signaling pathways genes is significantly correlated with the WHO level. Among them, SNAI1, SNAI2, BIRC5, CXCR4, TWIST1, and PDGFB are significantly increased in GBM. The WHO ratings are inversely related to the expression of ZEB1, ZEB2, and CXCL12.

Next, we investigated the status of IDH and 1p19q in EMT-associated proteins for various grades of gliomas. We found that MET, SNAI1, HGF, EGFR, ZEB1, SNAI2, PDGFB, CXCR4, TWIST1, and FZD4 were significantly differentially expressed between LGG with wildtype-IDH and LGG with mutant-IDH in CGGA dataset (Figure 1H). A similar pattern was observed for MET, SNAI1, and ZEB1 in GBM (Figure 1I). For the 1p19q status, SNAI1, PDGFB, HGF, TGFB1, EGF, CDH1, MET, IGF1, EGFR, ZEB2, and TWIST2 were significantly differentially expressed between LGG with codel-1p19q and LGG with Non-codel 1p19q (Figure 1J). In the LGG with mutant-IDH, the expression of SNAI1, PDGFB, TGFB1, EGF, CDH1, HGF, IGF1, EGFR, ZEB2, MET were associated with the status of 1p/19q codeletion (1p/19q codel) (Figure 1K). However, the expressions of these genes between GBM with IDH mutation in the CGGA dataset could not contrast, because the samples of GBMs with IDH mutation type were not enough (*n* = 35).

**Figure 2:**
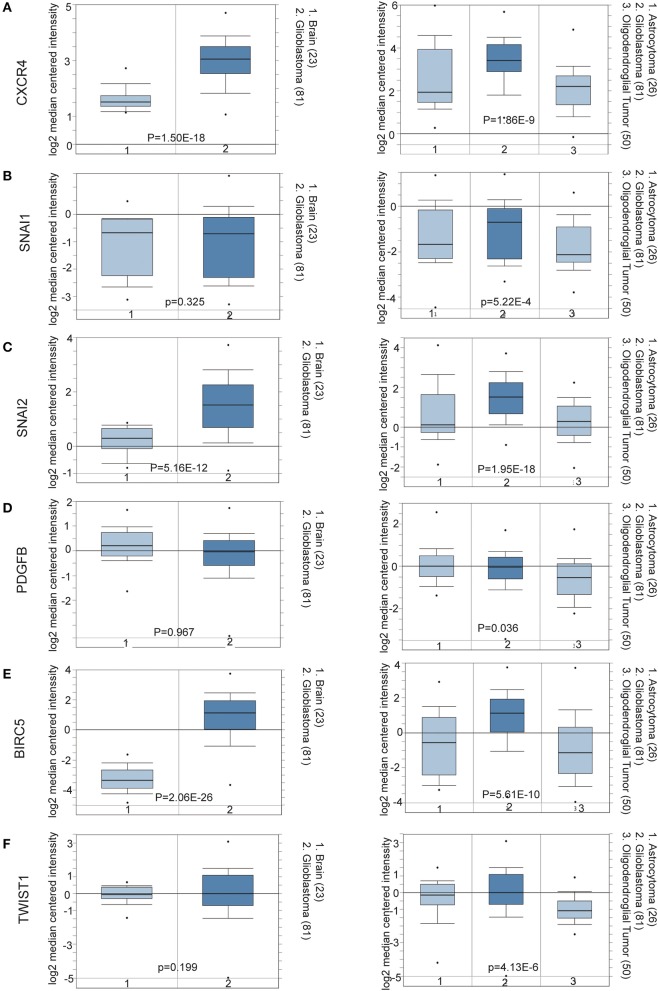
**(A–F)** Oncomine data mining analysis of SNAI1, SNAI2, BIRC5, CXCR4, TWIST1, and PDGFB mRNA levels in Sun Brain dataset between normal brain tissues and different pathological grade brain glioma tissues.

**Figure 3:**
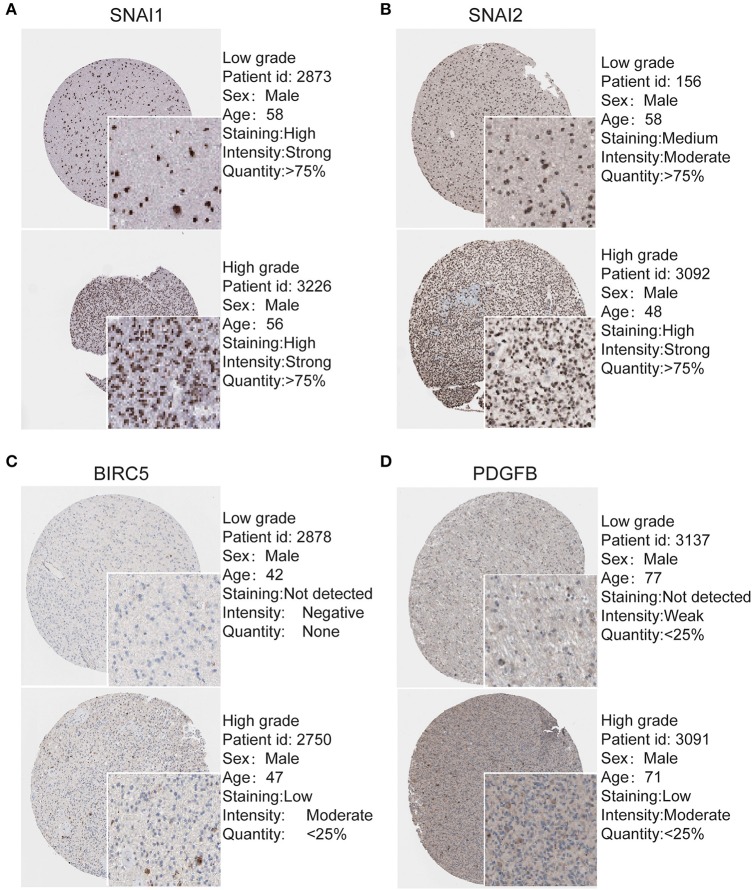
Validation of Most significantly EMT related genes in the turquoise module by The Human Protein Atlas database (IHC) **(A–D)**. There were no related IHC samples of CXCR4 and TWIST1 in the database. The translational expression level of the four EMT related genes was positively correlated with disease status as they were upregulated in glioma samples.

To determine the clinical significance of EMT related genes in patients with glioma, SNAI1, SNAI2, BIRC5, CXCR4, TWIST1, and PDGFB were selected, data mining and analysis were conducted to assess the protein expression based on the Oncomine database (Figures 2A–F). In addition, immunohistochemistry (IHC) datasets were retrieved from The Human Protein Atlas database which revealed the level of EMT-related proteins (No data found for CXCR4 and TWIST1) (Figures 3A–D).

### Consensus Clustering of EMT Related Genes Grouped Glioma Specimens Into Two Clusters With Different Pathological Characteristics and Clinical Outcomes

**Figure 4:**
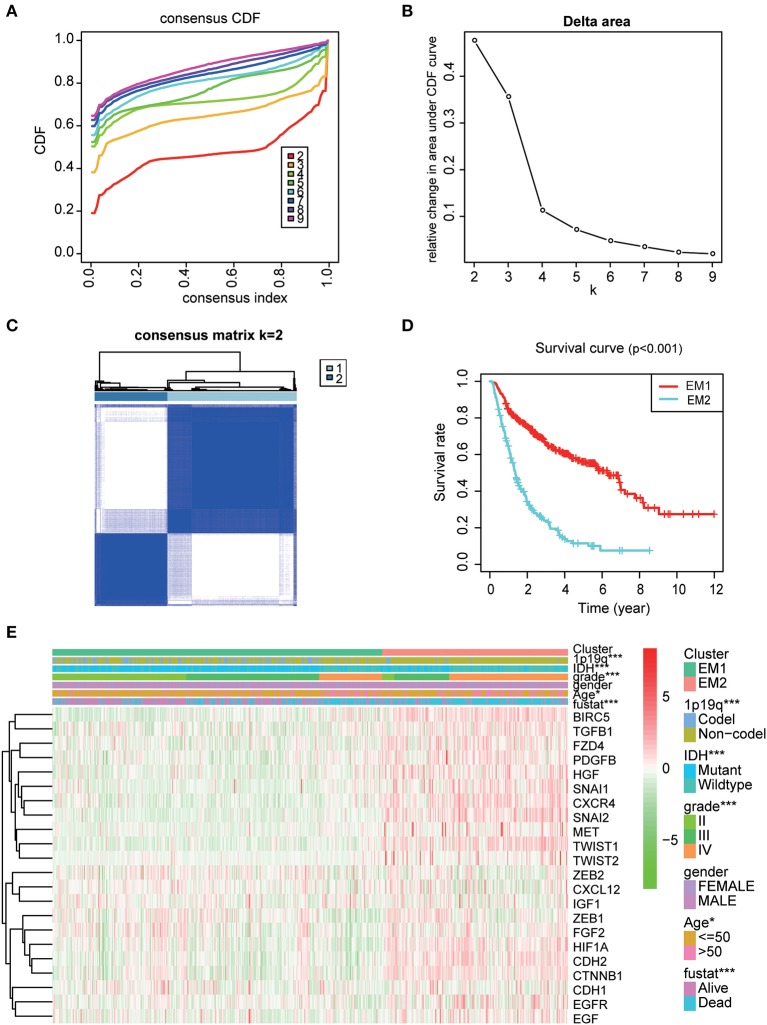
The distinction of clinicopathological features and OS of gliomas in the EM1/2 subgroups. **(A)** Consensus clustering cumulative distribution function (CDF) for *k* = 2–9. **(B)** Relative change in area under CDF curve for *k* = 2–9. **(C)** Correlation between the EM1 and EM2 subgroups. **(D)** Kaplan–Meier overall survival (OS) curves for 508 CGGA samples. **(E)** Heatmap and clinicopathologic features of the two clusters (EM1/2) defined by the EMT related genes consensus expression.

Using the similarities in the expression of EMT related genes, we choose the value of *k* = 2 (Figures 4A–C). The 508 glioma samples from the CGGA dataset were classified into two subgroups, the EM1 and EM2 groups (Figure S2). We further compared the clinicopathological features of the two subgroups. The EM1 subgroup mainly exhibited younger (*P* < 0.001) and lower grades (*P* < 0.001) at the time of diagnosis (Figure 4E). While the EM2 subgroup consisted of older and higher-grade glioma samples at the time of diagnosis. In addition, the overall survival (OS) of the EM2 subgroup was found to be shorter than that of the EM1 subgroup (Figure 4D). These results indicate that the two clusters were closely related to the malignancy glioma.

### The Categories Obtained From the Consistent Clustering Are Highly Related to the Degree of Malignancy of Gliomas

**Figure 5:**
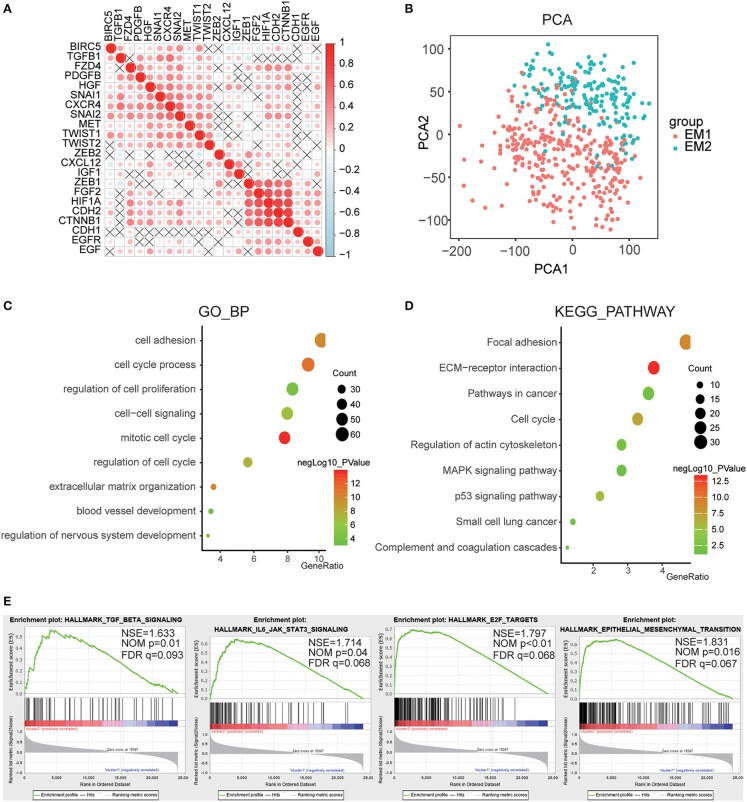
Interaction among EMT related genes and functional annotation of gliomas in EM1/2 subgroups. **(A)** Spearman correlation analysis of the 22 EMT related genes. **(B)** Principal component analysis of the total RNA expression profile in the CGGA dataset. **(C,D)** Functional annotation of the genes with different expression between two subgroups using GO terms of biological processes **(C)** and KEGG pathway **(D)**. **(E)** GSEA is used to annotate the malignant hallmarks of these genes with different expression levels between the two subgroups.

For a clearer understanding of the interactions of the 22 EMT related genes, we also analyzed their correlations. It was shown that the induction factor, transcription factors, signaling pathways genes of the same signaling pathway were significantly correlated (Figure 5A). The transcription profiles of EM1 and EM2 subgroups were compared using the principal component analysis (PCA), and the results suggested that there were significant differences between EM2 and EM1 (Figure 5B).

Subsequently, identified genes that were significantly differentially expressed between the two subgroups, and then analyzed their biological processes using the GO and KEGG pathways and annotated their functions. The results showed that the differentially expressed genes were enriched in the mitotic cell cycle, regulation of nervous system development, cell-cell signaling, cell adhesion, blood vessel development, etc. (Figure 5C). The KEGG pathway analysis suggested that ECM-receptor interaction, p53 pathway, pathways in cancer, and focal adhesion were significantly enriched (Figure 5D). Next, the GSEA was employed to determine the key signatures of malignant tumors. The result suggested that TGF-beta signaling, IL6/JAK/STAT3 signaling, E2F targets, epithelial-mesenchymal transition were related to malignancy (Figure 5E). These results indicate that the two categories strongly correlated with the malignancy of glioma cells.

### Prognostic Value of EMT Related Genes, and Risk Score Constructed Using Seven Selected Genes

**Figure 6:**
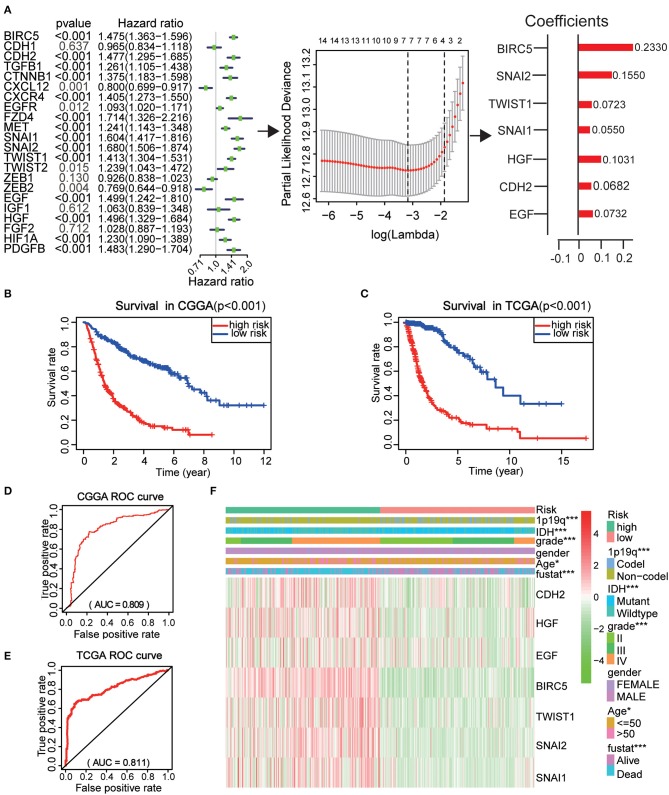
Risk signature with seven EMT related genes. **(A)** The process of using seven genes to building the signature. **(B,C)** Kaplan–Meier overall survival (OS) curves for patients in the CGGA **(B)** and TCGA **(C)** datasets assigned to high- and low-risk groups based on the risk score. **(D,E)** The ROC curves of the risk signature in CGGA 9 **(D)** and TCGA **(E)** datasets. **(F)** The heatmap of the seven EMT related genes in low- and high-risk gliomas. The distribution of clinicopathological features was compared between the low- and high-risk groups. *P < 0.05 and ***P < 0.001.

To better investigate the prognostic role of EMT related genes in gliomas, the expression of 22 genes in the CGGA dataset was assessed using univariate Cox regression analysis. The results showed that 14 of the 22 test genes were significantly associated with OS (*P* < 0.001) (Figure 6A). All the 14 genes, BIRC5, SNAI2, TWIST1, SNAI1, CXCR4, HGF, CDH2, PDGFB, MET, EGF, CTNNB1, FZD4, TGFB1, and HIF1A were found to be risky genes with HR >1.

Further, the CGGA datasets served as the training set and the minimum absolute contraction and selection operator (LASSO) Cox regression algorithm was applied. This revealed that the 14 genes were highly related to OS (*p* < 0.001) (Figures 6A,C). We then selected seven genes based on the minimum criteria to construct risk characteristics and used the coefficients derived from the LASSO algorithm to determine risk scores for each sample in the CGGA data set. The glioma patients in the CGGA (*n* = 508) dataset were classified into low and high-risk groups on the basis of the median risk score (Figure S3). We observed that there was a significant difference in OS between the two groups (*P* < 0.001) (Figure 6B). Next, we use the TCGA (*n* = 613) dataset as a validation dataset to calculate the risk scores. We observed a marked difference in OS between the two groups (*P* < 0.001) (Figure 6C). The ROC curve suggested that the risk score could accurately predict the survival rate of patients (Figures 6D,E).

Next, the risk score was applied to perform a heat map which showed that seven genes were highly expressed in patients with high and low risk in the CGGA dataset (Figure 6F). In both groups, WHO grade (*P* < 0.001), age (*P* < 0.05), IDH status (*P* < 0.001), 1p/19q codel status (*P* < 0.001), and EM1/2 subgroups were significant (*P* < 0.001). These analyses indicate that our selected genes and constructed risk prognostic models have good prognostic value.

### The Risk Score Has a Good Prognostic Performance and Is Closely Related to the Clinical Pathology of the Sample Population

**Figure 7:**
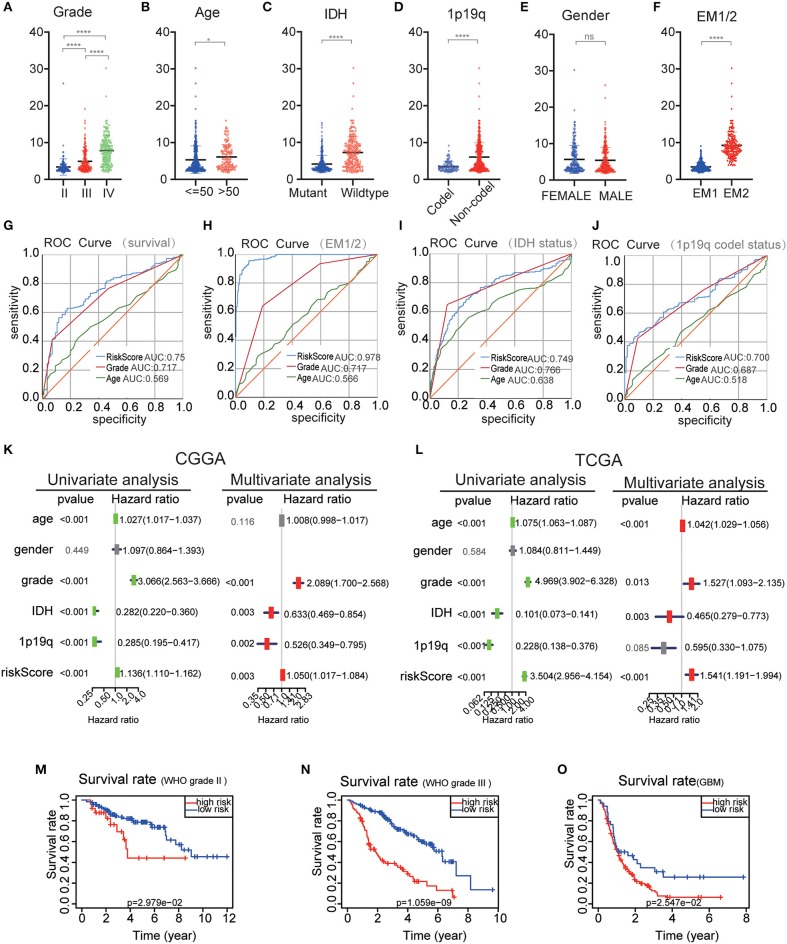
Comparison of risk scores and clinicopathological features between RM1/2 subgroups and differences in different grades of glioma OS. **(A–F)** Distribution of risk scores in the CGGA dataset stratified by WHO grade **(A)**, Age **(B)**, IDH status **(C)**, 1p/19q codel status **(D)**, gender **(E)**, and EM1/2 subgroups **(F)**. ns no significance, *P < 0.05 and ****P < 0.0001. **(G–J)** The predictive efficiency of the risk signature, WHO grade, and age on the survival rate **(G)**, EM1/2 subgroups **(H)**, IDH-mutant status **(I)**, and 1p/19q codel status **(J)** showed by ROC curves. **(K,L)** Univariate and multivariate Cox regression analyses of the association between clinicopathological factors (including the risk score) and overall survival of patients in the CGGA **(K)** and TCGA **(L)** datasets. **(M–O)** Kaplan–Meier survival curves with different WHO grade groups in the CGGA dataset.

The performance of the risk scores was tested for each clinicopathological feature. Significant differences were observed between the two groups in terms of WHO classification (*P* < 0.001), 1p / 19q codel status (*P* < 0.001) age (*P* < 0.05), and IDH status (*P* < 0.001) (Figures 7A–F). ROC curves showed that the risk scores could accurately predict the survival rate of patients with glioma (AUC = 0.750), glioma 1p/19q codel status (AUC = 0.700), glioma IDH-mutant status (AUC = 0.749), and EM1/2 subgroups (AUC = 0.978). In addition, we found that the risk score had a higher prediction accuracy in comparison with age and the WHO grade (Figures 7G–J). The multivariate Cox regression and univariate analyses performed on the CGGA datasets showed that the risk score, age and WHO grade were all related to OS (Figure 7K). Similar results were obtained in the validation dataset (TCGA dataset) (Figure 7L). These findings suggest that the risk score derived from EMT-related genes is an independent prognostic predictor of glioma patients. Next, we checked the prognostic potential of the risk model in various WHO grades. And OS was different between low-risk scores subgroup and high scores subgroup in WHO grade II, III gliomas and GBM (Figures 7M–O), It indicates that the risk score also has a good prognostic value in different grades of glioma.

## Discussion

Glioblastoma is considered to be a brain tumor with the highest malignancy and invasive nature (18, 27), and GBM has high levels of mortality. In this study, the expression of transcription factors and proteins related to EMT are reported to be highly related to the prognosis and malignancy of glioma. Two glioma subgroups EM1/2 were identified by consistent clustering on the basis of EMT-related genes. The EM1/2 subgroup not only affects disease progress and pathological characteristics, but it influences key signaling pathways, biological processes, and markers of malignancy in glioma. Moreover, we used seven selected EMT related genes to derive a prognostic risk factor, which classifies OSs in glioma patients into low and high-risk categories.

As EMT induction factors, SNAIL proteins can bind to E-box DNA sequences through their carboxy-terminal zinc-finger domains, thus activate the EMT programme during development, fibrosis and cancer (28, 29). ZEB1 and ZEB2 containing two zinc-finger domains that bind to E-boxes in regulatory regions, contributing to repression of epithelial genes as well as activation of mesenchymal genes (29, 30). HGF has been found to stimulate SNAIL2 or SNAIL1 expression to drive EMT (31, 32). EGF enhances the internalization of E-cadherin, and expression of TWIST or SNAIL1, thereby promoting EMT (33). Thus, we infer that the EMT induction factors have a close relationship with the EMT transcription factors, and play a key role in the EMT process. In the EMT process, ZEB2, ZEB1, SNAI2, and SNAI1 were reported to promote EMT markers expression, cell invasion, migration, and proliferation (8–11). CXCR4, BIRC5, CTNNB1, FZD4, and EGFR can promote invasion, adhesion and stimulate expression of EMT markers (13–17, 34). Snails participate in the growth and metastasis of papillary thyroid carcinoma (35), endometrioid endometrial carcinomas (36), and glioma (8). The level of SNAI2 correlates with poor prognosis in ovarian and breast tumors (37), and this is similar to SNAI2 expression in breast cancer that is partially differentiated (38). These findings suggest that different transcription factors and proteins together promote the occurrence of EMT, and these factors or proteins probably have different functions in different tumors.

We systematic analyzed the relationship of the EMT related genes in gliomas with different clinicopathological features. As EMT induction factor, transcription factors, the expression of SNAI1, SNAI2, TWIST1, and PDGFB was significantly higher in high-grade, elderly glioma patients, IDH-wildtype, 1p19q non-codel glioma patients. Whereas ZEB1 and ZEB2 expression were highly expressed in low-grade, IDH-Mutant, 1p19q codel and younger glioma patients, indicating that the potential function of these genes in glioma malignancies. For signaling pathways genes, the expression of CXCR4, BIRC5, CTNNB1, FZD4, and MET were significantly increased in high-grade gliomas. These datasets indicate that the expression of EMT-associated genes is closely related to the malignant clinicopathological features in gliomas. In this study, we also analyzed the biological processes and signaling pathways of EMT and malignant progression of glioma. In addition to increasing the transcription factors required for EMT, TGFβ also enhances the transcriptional activities of these factors. Moreover, TGFβ promotes Smad-independent signaling thereby activating PI3K/Akt-MAPK or mTOR1 signaling (5, 39, 40), TGFB1 enhances epithelial plasticity which is likely to progress to EMT in carcinomas (41, 42). SDF-1/CXCR4 signaling is a positive regulator of EMT as it stimulates Wnt/β-catenin, PI3K/Akt, or MEK/ERK pathway (43). Subsequently, Wnt/β-catenin pathway enhances the expression of Snail, TWIST and ZEB1 which regulate the EMT process, causing an increase in the invasion and migration of glioma cells (14). In the present study, a switch from E- Cadherin (CDH1) to N-Cadherin (CDH2) was identified in many pathways. TWIST promotes N-cadherin and decreases E-cadherin expression, leading to tumor cell chemoresistance, metastasis, invasion, and migration in different types of cancer (44–46). ZEB and SNAIL bind to the E-Box motif in the promoter region of the CDH1 gene, leading to the inhibition of transcription (11, 42). In this study, we compared CDH1 and CDH2. We found that these factors increased the expression of cadherins, especially CDH4 and CDH2 (20). Our results also reveal that EMT genes are correlated with cellular processes, e.g., cell adhesion, cell-cell signaling, mitotic cell cycle, blood vessel development, regulation of nervous system development, and signaling pathways, including cell cycle, p53 signaling pathway, focal adhesion, and ECM-receptor interaction. Here, seven EMT related genes were used to design a prognostic signature for glioma based on the EMT associated induction factor, transcription factors, and signaling pathways genes. The risk score showed high prognostic performance for GBM gliomas in the CGGA dataset and gliomas categorized by 2016 WHO grades II and III.

We have learned that EMT can increase the resistance of malignant cells to multiple treatments (47). However, since EMT-related transcription factors cannot be suppressed by conventional pharmacology and antibody targeting methods, no effective method has been developed to suppress the EMT phenomenon, thereby inhibiting tumor invasion and metastasis (48). Kudo-Saito et al. have found that injecting Snail-specific small interfering RNA (siRNA) into the melanoma can inhibit tumor growth and metastasis, promote the number of tumor-specific tumor-infiltrating lymphocytes increase and enhance the systemic immune response in mice (49). And the interaction between immune microenvironment and cancer cells is important for tumor progression (50). Therefore, the potential of molecularly targeted immunotherapy to treat these EMT-related transcription factors may be promising.

Looking forward, studies aimed at revealing the exact mechanisms and genes that regulate EMT in GBM are advocated. This will enable the design of new drugs or strategies for managing gliomas (18).

## Conclusion

In conclusion, this study presents the expression, function, and prognostic potential of EMT related genes in gliomas. Our findings are important for future explorations into the role of the EMT process in gliomas.

## Data Availability Statement

We obtained the Clinicopathological data sets from CGGA (http://www.cgga.org.cn/) and TCGA (http://cancergenome.nih.gov/).

## Author Contributions

XZ and KH designed the research. CT, KH, JS, QH, and KL performed the literature search, created tables, and figures, and were involved in manuscript writing and proofreading. XZ and KH supervised the research and critically read the draft manuscript. All authors proofread and approved the final manuscript.

### Conflict of Interest

The authors declare that the research was conducted in the absence of any commercial or financial relationships that could be construed as a potential conflict of interest.
