# Supplementary figures and images for: Genomics and Prognosis Analysis of Epithelial-Mesenchymal Transition in Glioma
Source: Front Oncol. 2020 Feb 21;10:183. doi: 10.3389/fonc.2020.00183 (PMC7047417; doi:10.3389/fonc.2020.00183)

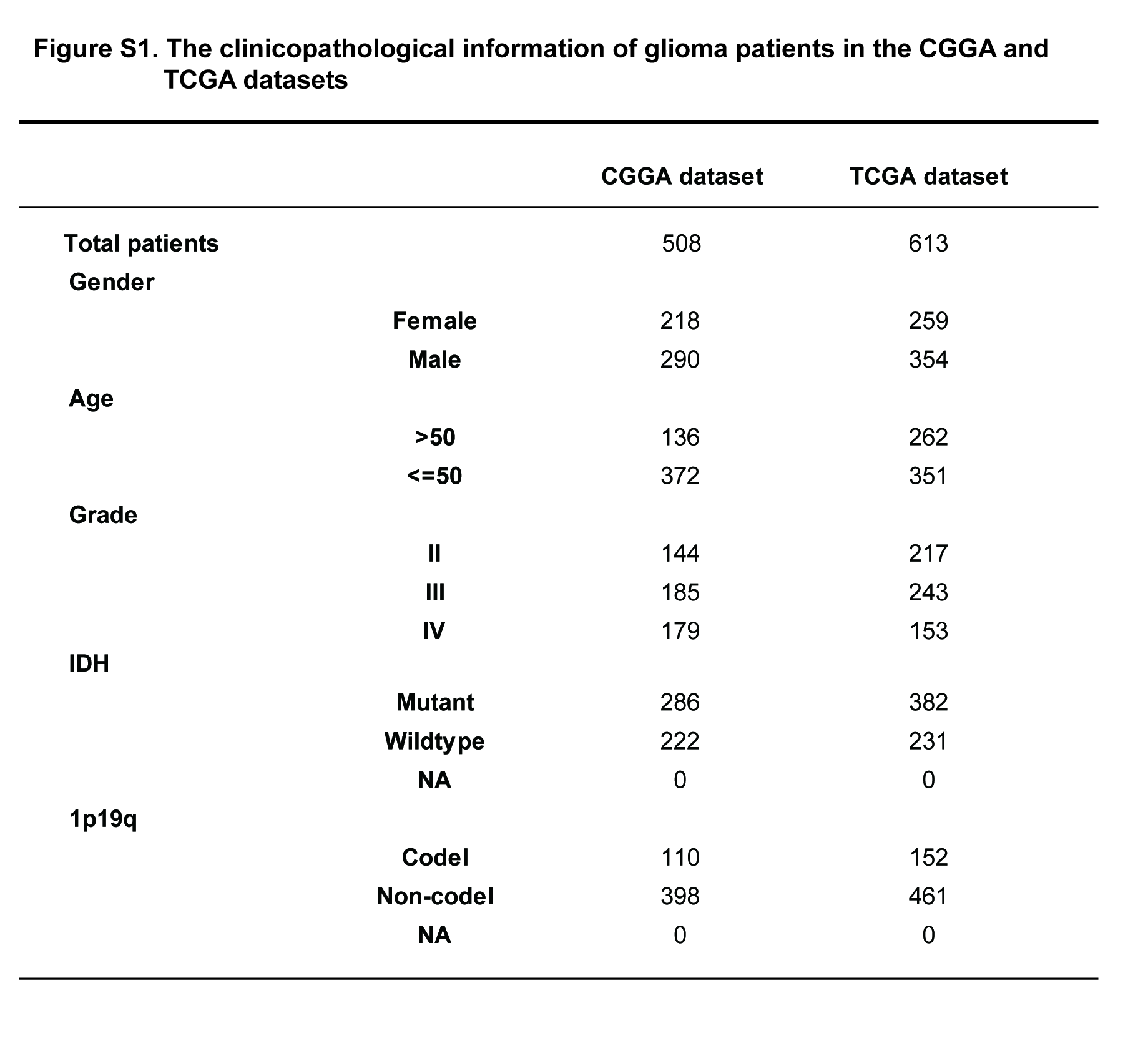

Supplement: Supplementary file 1 [file Image_1.TIF]

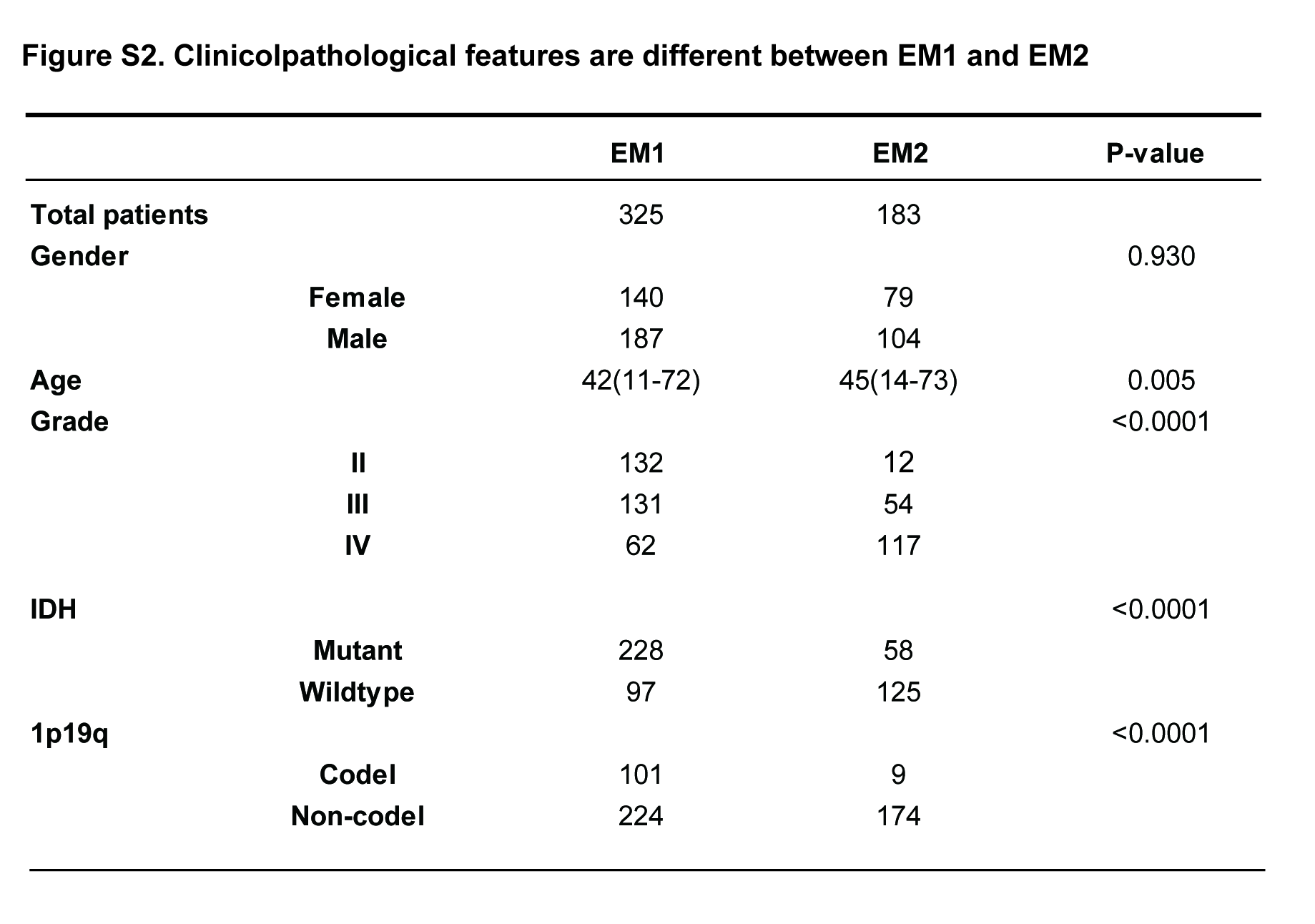

Supplement: Supplementary file 2 [file Image_2.TIF]

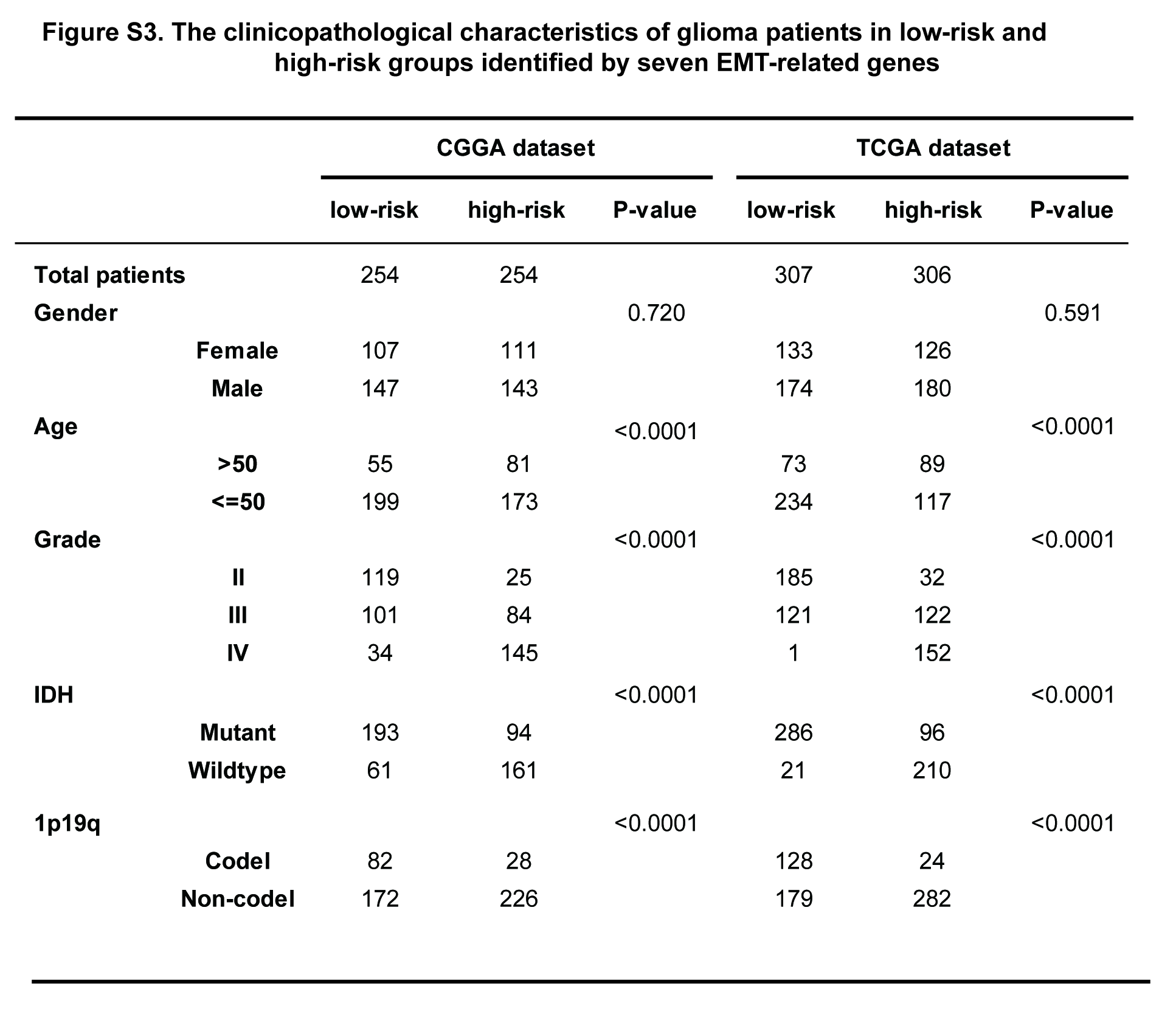

Supplement: Supplementary file 3 [file Image_3.TIF]
