# Supplementary material for: Genomics and Prognosis Analysis of Epithelial-Mesenchymal Transition in Glioma
Source: Front Oncol. 2020 Feb 21;10:183. doi: 10.3389/fonc.2020.00183 (PMC7047417; doi:10.3389/fonc.2020.00183)

Supplementary Material

# Supplementary Data

Non

# Supplementary Figures and Tables

## Supplementary Figures


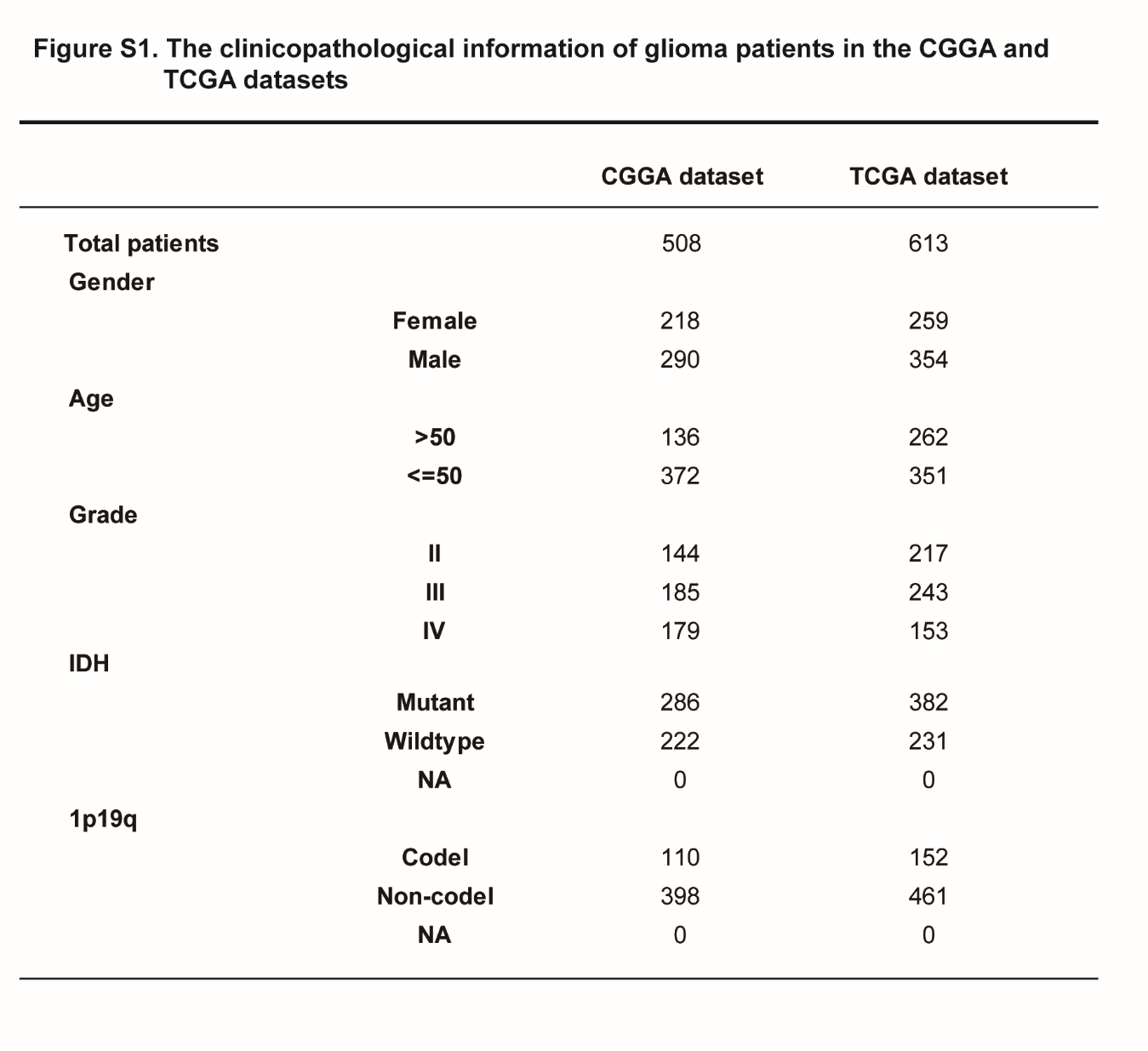


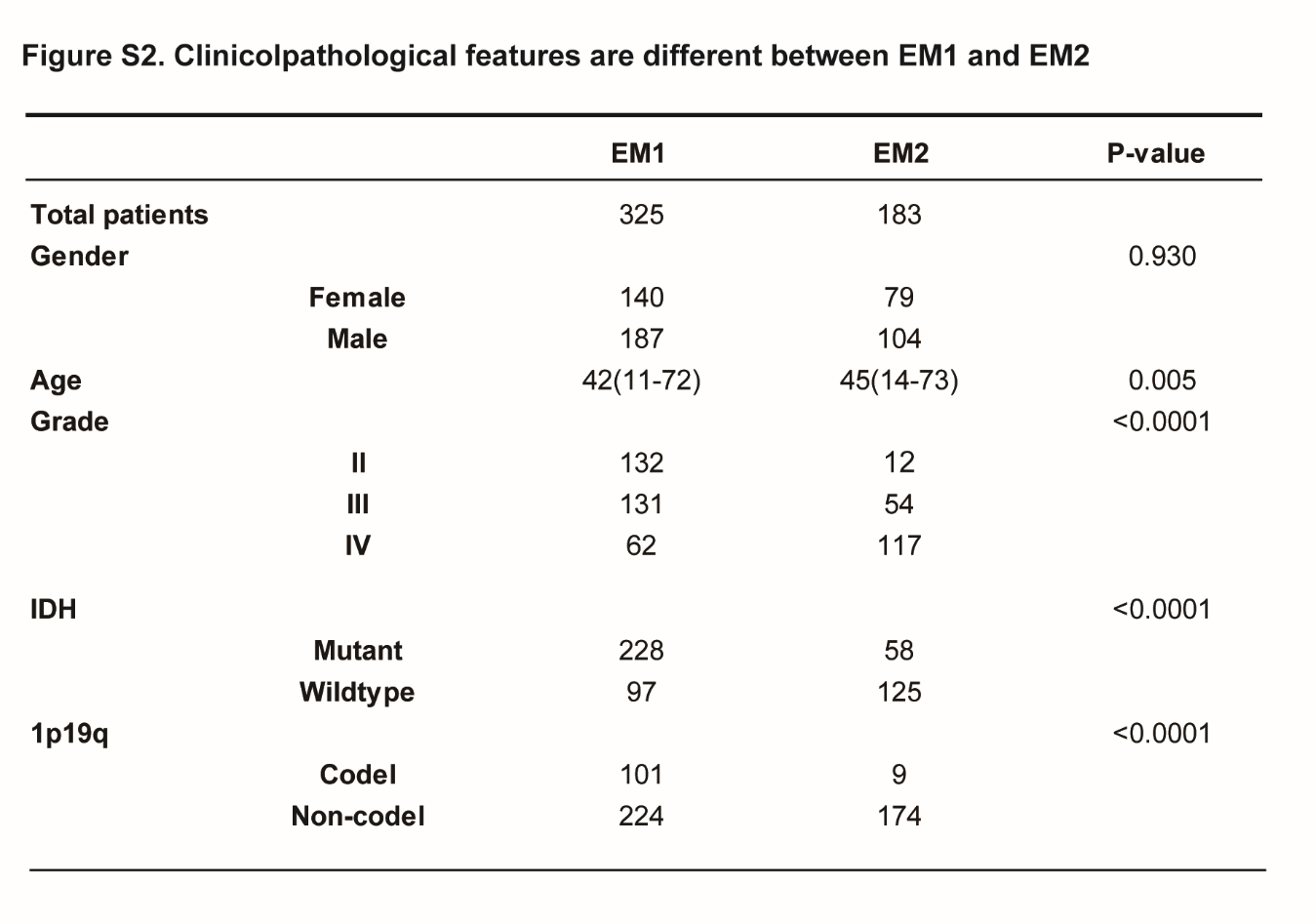

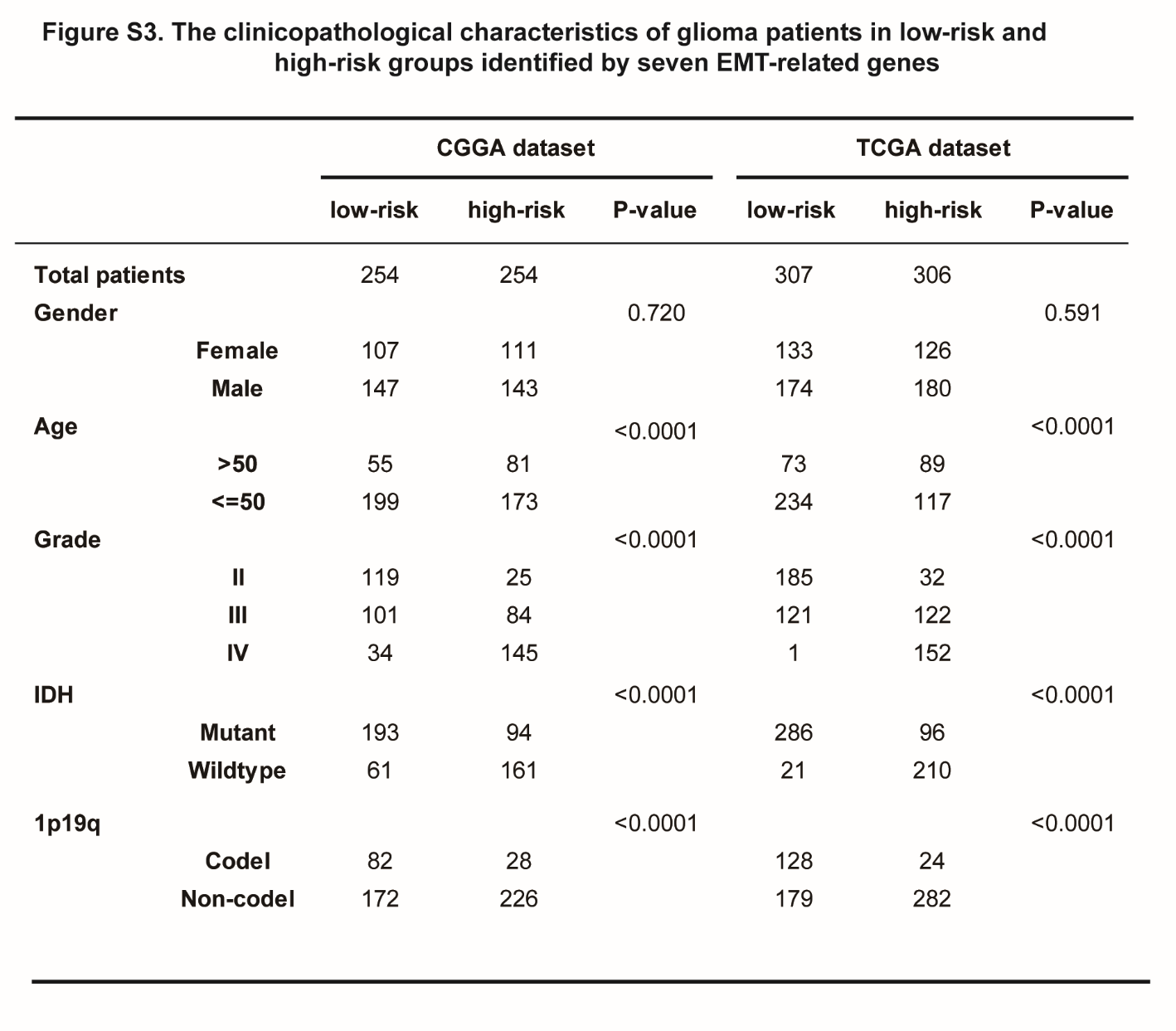

Supplement: Supplementary file 4 [file Table_1.DOCX]
